# Research Progress on the Mechanism of Histone Deacetylases in Ferroptosis of Glioma

**DOI:** 10.3389/or.2024.1432131

**Published:** 2024-08-12

**Authors:** Meng Ma, Xifeng Fei, Dongyi Jiang, Hanchun Chen, Xiangtong Xie, Zhimin Wang, Qiang Huang

**Affiliations:** ^1^ Department of Neurosurgery, Dushu Lake Hospital Affiliated to Soochow University, Suzhou, China; ^2^ Department of Neurosurgery, Suzhou Kowloon Hospital, Shanghai Jiaotong University School of Medicine, Suzhou, China; ^3^ Department of Neurosurgery, The Second Affiliated Hospital of Soochow University, Suzhou, China

**Keywords:** ferroptosis, histone deacetylases, histone deacetylase inhibitors, glioma, targeted therapy

## Abstract

Glioma is the most prevalent primary malignant tumor of the central nervous system. While traditional treatment modalities such as surgical resection, radiotherapy, and chemotherapy have made significant advancements in glioma treatment, the prognosis for glioma patients remains often unsatisfactory. Ferroptosis, a novel form of programmed cell death, plays a crucial role in glioma and is considered to be the most functionally rich programmed cell death process. Histone deacetylases have emerged as a key focus in regulating ferroptosis in glioma. By inhibiting the activity of histone deacetylases, histone deacetylase inhibitors elevate acetylation levels of both histones and non-histone proteins, thereby influencing various cellular processes. Numerous studies have demonstrated that histone deacetylases are implicated in the development of glioma and hold promise for its treatment. This article provides an overview of research progress on the mechanism by which histone deacetylases contribute to ferroptosis in glioma.

## Introduction

Glioma is the most prevalent primary malignant tumor of the central nervous system. While traditional treatment modalities such as surgical resection, radiotherapy, and chemotherapy have shown progress in glioma management, patient prognosis remains unsatisfactory due to the extensive phenotypic heterogeneity and plasticity of the tumor [1, 2]. The heterogeneity emerges as a consequence of the genetic diversity of glioma cells, and its plasticity is manifested in the tumor cells’ capacity to adapt to environmental alterations [2]. Although immune treatment strategies such as checkpoint blockade (ICB), immune modulators, and CAR-T cell therapy have shown potential in other tumors, the immune characteristic of glioma being a “cold tumor” limits the effect of immune therapy. Therefore there is an urgent need to find ways to enhance the effectiveness of glioma immunotherapy [3].

Ferroptosis, as a novel form of programmed cell death, is the most functionally diverse process in glioma and is involved in the immunological microenvironment of glioma, offering new research avenues for glioma treatment [4, 5]. Acetylation, a crucial post-translational modification, orchestrates cellular growth and development through modulating gene transcription, with the fine balance of histone acetylation levels being precisely regulated by the coordinated actions of histone acetyltransferases (HATs) and histone deacetylases (HDACs) [6, 7]. Histone deacetylase inhibitors (HDACi) are currently being used alone or in combination to treat certain tumors [8–12]. The acetylation status of histones affects the process of ferroptosis in tumor cells. By using HDACi to decrease HDAC activity, the induction of ferroptosis in adrenocortical cancer cells can be accelerated, inhibiting tumor growth and proliferation. However, the specific mechanism underlying this process is not yet fully understood [13]. Therefore, this article primarily reviews research progress on the mechanism of HDACs in the process of ferroptosis in glioma.

## A New Perspective in Glioma Research: Ferroptosis and Glioma

### Ferroptosis

Iron, an essential trace element in the human body, plays a crucial role in vital activities such as DNA synthesis, ATP production, and mitochondrial metabolism [14]. Maintaining iron homeostasis is important for the normal progression of life processes. Disruption of iron homeostasis can affect normal physiological processes and lead to various diseases such as tumors, aging, and infections. Excessive iron within cells can lead to the production of reactive oxygen species (ROS) through the Fenton reaction, triggering oxidative stress responses [15, 16]. Conversely, iron deficiency can impact cell structure in the body and result in diseases such as anemia, weakened immunity, and digestive disorders and so on [17–19].

Cells, as the fundamental units of life, are confronted with the challenge of iron overload during their growth and development. This involves how cells respond to various oxidative stress responses, a process that ultimately determines the fate of the cell. Dixon et al. have identified a novel form of programmed cell death known as ferroptosis. Ferroptosis is characterized by lipid peroxidation on the cell membrane, leading to an accumulation of iron within the cell as a primary manifestation [20]. Unlike traditional forms of apoptosis (such as apoptosis, pyroptosis, etc.), ferroptosis is distinguished by mitochondrial shrinkage and a reduction in the number of mitochondrial cristae [21]. Additionally, certain conditional factors such as synthesis and peroxidation of polyunsaturated fatty acid phospholipids (PUFA-PLs), disorder in iron metabolism, and mitochondrial dysfunction are necessary for the occurrence of ferroptosis [22]. Cells possess four defense systems against ferroptosis: GPX4-GSH system, FSP1-CoQH2 system, DHODH-CoQH2 system, and GCH1-BH4 system [21]. When these defense systems fail to effectively buffer against ferroptosis, it results in cellular demise. These mechanisms offer potential pathways for intervening in ferroptosis.

Ferroptosis is a form of cell death associated with various pathological processes, particularly in tumors, neurodegenerative, and inflammatory diseases [23–30]. It holds great potential in tumor therapy. Tumor cells have a higher metabolic rate and require more oxygen, leading to the production of reactive oxygen species. This characteristic makes tumor cells more susceptible to ferroptosis, making it an important mechanism for tumor suppression. However, tumor cells can counteract ferroptosis by limiting the synthesis and peroxidation of PUFA-PLs [31], restricting the availability of unstable iron [32], and upregulating cellular defense systems against ferroptosis [33]. These regulatory responses play a key role in the survival of tumor cells and may pose as obstacles to treatment. The induction of ferroptosis not only inhibits tumor growth but may also damage non-tumor cells. Therefore, precise control over the induction of ferroptosis is currently a focus in research efforts aimed at primarily affecting tumor cells while protecting normal cells, especially immune system-related cells. In this way, ferroptosis is expected to be developed as a new therapeutic strategy that effectively inhibits tumors while preserving the body’s immune function—a significant development in treating various diseases.

### The Impact of Ferroptosis on the Blood-Brain Barrier

The blood-brain barrier (BBB) is a critical protective mechanism of the central nervous system, regulating the passage of substances into and out of the brain while maintaining its stability. Composed of endothelial cells, pericytes, astrocytes, microglia, and neurons among others, the interaction between these cells is essential for BBB function [34]. The unique characteristics of BBB endothelial cells include a flat appearance, tight junctions, and a higher number of mitochondria which contribute to regulating and maintaining BBB function [35]. Lipids are a vital component of cell membranes and are particularly abundant in the central nervous system [36]. Lipid peroxidation can alter cell membrane fluidity and permeability, impacting cell structure and function. Cyclooxygenase (COX), cytochrome P450 (CYP), and lipoxygenase (LOXs) are the three primary lipid oxidases involved in iron-dependent lipid peroxidation [37]. Iron accumulation and lipid peroxidation are linked to BBB dysfunction; understanding the relationship between ferroptosis and BBB dysfunction offers new avenues for treating central nervous system diseases.

The p53 protein is responsible for maintaining the permeability and integrity of BBB cells by reducing the level of lipid peroxidation [38]. Dysfunction of the BBB is among the early pathophysiological changes observed in neurodegenerative diseases and brain injuries, such as stroke, traumatic brain injury (TBI), Alzheimer’s disease (AD), and Parkinson’s disease (PD) [39–41]. The BBB serves as the primary barrier for iron to enter the brain and plays a critical role in maintaining brain iron balance. Research has indicated that iron chelators can protect brain microvascular endothelial cells from toxicity and functional damage, thereby preserving endothelial cell stability [42]. Excessive accumulation of iron can induce the expression of matrix metalloproteinases (MMPs), which in turn leads to degradation of vascular basement membrane components, causing damage to the BBB [43, 44]. Additionally, reactive oxygen species (ROS) can impact BBB function by altering intracranial vascular tension, increasing platelet aggregation, and endothelial cell permeability; ultimately resulting in focal lesions on the endothelial cell membrane [45]. The interaction between ROS and MMPs may ultimately lead to dysfunction of the BBB [46]. Although initial progress has been achieved in comprehending the connection between BBB dysfunction and ferroptosis, the current literature is still inadequate in uncovering the molecular mechanism of their interaction, and there is a shortage of conclusive experimental data to substantiate it. Additionally, existing studies have not comprehensively expounded the universality and applicability of BBB dysfunction in various pathological conditions. In light of this, the research on the BBB is particularly crucial in the process of ferroptosis. This not only enriches our understanding of the pathological mechanism of central nervous system diseases but also holds significant guiding significance for the development of new treatment strategies and the improvement of treatment efficacy.

### The Role of Ferroptosis in Glioma

In recent years, ferroptosis has garnered widespread attention in the field of tumor research, particularly demonstrating potentially significant effects in glioma and the tumor microenvironment [5, 47]. Glioma is a prevalent malignant tumor of the central nervous system, and its treatment difficulty stems from the resistance of tumor cells to traditional radiotherapy and chemotherapy, as well as the limitation of drug delivery imposed by the blood-brain barrier. The discovery of ferroptosis offers a new perspective and potential treatment strategy for glioma.

Due to their rapid proliferation and high metabolic activity, glioma cells have a high demand for iron, making them potentially vulnerable in terms of iron metabolism. The induction of ferroptosis is closely related to the disorder of iron metabolism. Therefore, regulating iron metabolism or directly inducing ferroptosis may become a new approach to the treatment of glioma. Studies have shown that certain iron chelators and ferroptosis inducers can increase the sensitivity of glioma cells to radiotherapy and chemotherapy, thereby enhancing the therapeutic effect [48, 49]. ATF3 contributes to brucine-triggered glioma cell ferroptosis via promotion of hydrogen peroxide and iron [50]. High levels of NRF2 sensitize temozolomide-resistant glioblastoma cells to ferroptosis via ABCC1/MRP1 upregulation [51]. PRMT1 driven PTX3 regulates ferritinophagy in glioma [52]. Gastrodin Inhibits H2O2-Induced Ferroptosis through Its Antioxidative Effect in Rat Glioma Cell Line C6 [53]. Ibuprofen induces ferroptosis of glioblastoma cells via downregulation of nuclear factor erythroid 2-related factor 2 signaling pathway. Anticancer Drugs [54]. Pseudolaric acid B triggers ferroptosis in glioma cells via activation of Nox4 and inhibition of xCT [55]. TRIM7 modulates NCOA4-mediated ferritinophagy and ferroptosis in glioblastoma cells [56]. Targeting NQO1/GPX4-mediated ferroptosis by plumbagin suppresses *in vitro* and *in vivo* glioma growth [57]. In addition, the induction of ferroptosis not only involves inducing ferroptosis in glioma cells but may also affect the iron metabolism of related cells in the tumor microenvironment. The microenvironment of glioma is usually rich in factors that promote tumor growth, and promoting or inhibiting ferroptosis may change the balance of these factors, thus affecting the growth and invasion of the tumor. For instance, SLC1A5 enhances malignant phenotypes through modulating ferroptosis status and immune microenvironment in glioma [58]. Within the glioma microenvironment, the ferroptosis inducer erastin has been shown to enhance the polarization of macrophages towards the M2 phenotype. Conversely, the ferroptosis inhibitor ferrostatin-1 has been found to increase the number of M1-like macrophages and decrease the number of M2-like macrophages in the glioma microenvironment, thereby exerting an inhibitory effect on glioma development [4]. Additionally, an innovative NRF2 nano-modulator has demonstrated its ability to induce ferroptosis in lung cancer, leading to a shift in M2 macrophage polarization towards M1 and enhancing anti-tumor immunity [59]. The critical factors influencing ferroptosis in glioma are presented in Table 1. However, there are challenges associated with utilizing ferroptosis for treating glioma. Firstly, a key issue is how to precisely induce ferroptosis in glioma cells without causing damage to normal cells. Secondly, effective drug delivery to the tumor site is hindered by the presence of the blood-brain barrier. As a result, researchers are exploring various strategies such as developing new ferroptosis inducers, leveraging nanotechnology to improve drug penetration, and combining other treatment methods for comprehensive care.

**TABLE 1:** Critical factors of ferroptosis in glioma.

Critical factors classification	Critical factors	Mechanism
Regulation of Transcription Factors	NRF2	NRF2 sensitize temozolomide-resistant glioblastoma cells to ferroptosis via ABCC1/MRP1 upregulation [51]
Regulation of Transcription Factors	ATF3	ATF3 contributes to brucine-triggered glioma cell ferroptosis via promotion of hydrogen peroxide and iron [50]
Regulation of Transcription Factors	p53	The extent of acetylation of p53 impacts the expression of ferroptosis-related genes, potentially facilitating ferroptosis through the depletion of SLC7A11 levels [60–62]
Regulation of iron metabolism	SLC7A11	SLC7A11 is involved in the exchange of cystine and glutamate between the intracellular and extracellular compartments, and influences glutathione (GSH) biosynthesis [63–65]
Signaling pathways governing	NF-κB,STAT3,STAT6	These pathways regulate the ferroptosis process through the expression and activity of iron metabolism-related proteins [66–68]
The antioxidant defense mechanism	Antioxidant enzymes (e.g., SIRT3, GPX4)	GPX4 safeguards cell membranes against oxidative damage and sustains the oxidation-reduction equilibrium of cells through the reduction of lipid peroxide and the elimination of ROS [56, 68, 69]
Regulation of iron transportation	SLC1A5	Regulate the immune microenvironment, the state of iron transport in GBM, and enhance the malignant phenotype [58]

Note. NRF2, Nuclear factor erythroid 2-related factor 2; ATF3, Activating Transcription Factor 3; p53, Tumor Protein p53; SLC7A11, Solute Carrier Family 7 Member 11; NF-κB, nuclear factor kappa-B; STAT3, Signal transducer and activator of transcription 3; STAT6, Signal transducer and activator of transcription 6; SIRT3, Sirtuin 3; GPX4, Glutathione Peroxidase 4; SLC1A5, Solute Carrier Family 1 Member 5.

## A New Target for Glioma Treatment: Histone Deacetylases

The level of histone acetylation is jointly regulated by HATs and HDACs, which influence the structure of chromatin and gene transcription. There are five families of HATs, including p300/CBP, GNAT, SRC, MYST, and TAFII250. These enzymes catalyze the transfer of acetyl-coenzyme A to lysine residues, promoting chromatin relaxation and gene activation [70, 71]. The function of HDACs is opposite to that of HATs; HDACs catalyze deacetylation by hydrolyzing the acetyl groups on lysine residues, leading to a decrease in histone acetylation levels, which enhances the interaction between histones and DNA. This results in a more condensed chromatin state and suppresses gene transcription [72]. The HDAC family is diverse, with 18 members divided into four classes. Class I HDACs (HDAC1, HDAC2, HDAC3, and HDAC8) are related to the yeast RPD3 deacetylase and are primarily involved in cell proliferation, differentiation, DNA damage response, and tissue development in the nucleus. Class II HDACs can be further divided into two subclasses: Class IIa (HDAC4, HDAC5, HDAC7, and HDAC9) and Class IIb (HDAC6 and HDAC10), which are homologous to the yeast Hda1 deacetylase. Class III HDACs (SIRT1, SIRT2, SIRT3, SIRT4, SIRT5, SIRT6, and SIRT7) share sequence similarity with the yeast Sir2 protein. Class IV HDAC (HDAC11) has sequence similarity with Class I and Class II proteins [73]. The balance of these enzymes is crucial for maintaining cellular functions.

In glioma, the abnormal activity of HDACs is associated with tumor growth, invasiveness, and drug resistance [74–77]. HDACs regulate the expression of related genes by modulating the acetylation level of histones. These genes may involve iron metabolism and antioxidant defense systems, thereby indirectly affecting the process of ferroptosis. Therefore, HDAC inhibitors (HDACIs), as potential therapeutic agents, have become a new direction in the treatment of glioma. Understanding the role and expression patterns of HDACs in different subtypes of glioma will help develop more precise and personalized treatment plans.

## The Impact of Acetylation on Ferroptosis

Acetylation, as an epigenetic modification, orchestrates cellular processes by influencing the intricate structure of chromatin, thereby altering protein function, stability, and subcellular localization. The impact of acetylation on ferroptosis primarily manifests through its modulation of the antioxidant defense mechanism, regulation of transcription factors, and control over iron metabolism-related proteins and signaling pathways.

### Acetylation Affects the Antioxidant Defense Mechanism

Antioxidant enzymes are a critical component of the cellular antioxidant defense system, protecting cells from oxidative damage by neutralizing reactive oxygen species (ROS). Acetylation has been shown to modulate the activity of these antioxidant enzymes. For example, SIRT3 enhances the activity and stability of antioxidant enzymes through deacetylation, thereby enhancing the cell’s antioxidant capacity [69]. The cystine/glutamate antiporter SLC7A11 (also known as xCT) plays a significant role in ferroptosis by facilitating the exchange of cystine and glutamate across the cell membrane. This process imports cystine for glutathione (GSH) biosynthesis and supports antioxidant defense. SLC7A11 is also overexpressed in various human cancers [63]. Recent studies indicate that overexpression of SLC7A11 partially promotes tumor growth by inhibiting ferroptosis. Additionally, it has been observed that knockdown of the TP53 gene increases transcriptional levels of SLC7A11; conversely, overexpression of TP53 downregulates SLC7A11 expression and cystine release. These findings suggest a negative correlation between p53 expression and SLC7A11 expression, indicating that p53 may suppress the activity of SLC7A11 directly or indirectly promote ferroptosis to inhibit glioma cell growth [60]. However, high overexpression of SLC7A11 can unexpectedly inhibit tumor metastasis [78], highlighting the dynamic nature of ferroptosis. Although there is currently no direct evidence supporting acetylation’s regulation on SLC7A11, it is possible that acetylation may influence its gene expression by affecting transcription factors such as p53. This underscores the need for further comprehensive study into understanding how different regulatory mechanisms impact ferroptosis dynamics.

### Acetylation Regulates the Activity of Transcription Factors

Acetylation plays a critical role in regulating the activity and DNA-binding capacity of transcription factors. An example of this is the impact of acetylation on p53, which in turn affects the expression of genes related to ferroptosis. As a crucial tumor suppressor, p53 regulates various biological processes such as cell proliferation, DNA repair, apoptosis, and autophagy [79]. The pioneering work of researchers like Wei Gu et al. has confirmed that the p53 protein can undergo acetylation modification through a process involving multiple proteins and complex regulation [80]. While there is functional redundancy in the acetylation sites of p53, meaning that loss of one or more sites can be compensated by acetylation at other sites, it has been observed that loss of the eight primary acetylation sites in human p53 (the 8 KR mutant) results in diminished transcriptional activity and hinders its ability to induce cell cycle arrest and/or apoptosis [61]. This finding challenges previous notions about functional redundancy and has sparked significant interest in understanding the functional role of p53 acetylation sites.

The acetylation modification also plays an important role in regulating not only the overall transcriptional activity but also the site-specific transcriptional selectivity of p53. This precise control allows for regulation of key biological processes such as cell cycle arrest, apoptosis, senescence, autophagy, and metabolism [81–83]. Furthermore, p53 has been shown to inhibit glioma growth by inducing ferroptosis [84], while a novel histone deacetylase inhibitor called MPT0B291 has demonstrated efficacy in suppressing glioma growth both *in vitro* and *in vivo* by increasing the level of p53 acetylation [62]. In conclusion, it is evident that the acetylation state of p53 is crucial for its function in inhibiting tumor metabolism and inducing ferroptosis.

### Acetylation Regulates Iron Metabolism-Related Proteins and Cellular Signaling Pathways

Acetylation plays a significant role in the regulation of iron metabolism-related proteins. Hepcidin, a small peptide secreted by the liver, is crucial for maintaining systemic iron homeostasis. The downregulation of hepcidin expression is associated with the epigenetic regulation of HDAC3. Hepcidin controls systemic iron levels by inhibiting intestinal iron absorption and recycling [85]. Acetylation modification can participate in the regulation of various signaling pathways, which may impact the process of ferroptosis. STAT6 has been found to inhibit ferroptosis and alleviate acute lung injury by competitively binding to CREB-binding protein, a key acetyltransferase that regulates the p53/SLC7A11 pathway [66]. The NF-κB and STAT3 pathways also play important roles in regulating ferroptosis. Activation of the NF-κB pathway can increase the expression of LCN2, an iron-sequestering cytokine that may be related to tumor resistance to ferroptosis-inducing drugs [67]. Additionally, STAT3 can promote GPX4 transcription by binding to its promoter region. GPX4 is an essential antioxidant enzyme that protects cells from ferroptosis. Therefore, inhibition of STAT3 may reduce GPX4 expression and promote ferroptosis [68]. In summary, acetylation can influence the process of ferroptosis through multiple mechanisms, which may play a pivotal role in tumor development and treatment. Future research needs to delve deeper into these mechanisms and explore the specific role of acetylation in ferroptosis to develop more effective therapeutic strategies.

## The Role of HDAC Inhibitors in Ferroptosis in Glioma

HDACi influence various cellular processes by suppressing HDAC activity, leading to increased acetylation levels of histones and non-histone proteins within the cell. This ultimately exerts anti-tumor effects by inducing histone acetylation and regulating gene transcription [86]. While research on HDACi in the context of ferroptosis is still relatively scarce, as our understanding of the role of acetylation in ferroptosis deepens, researchers are beginning to focus on the role of HDACi in this process.

As epigenetic regulatory molecules, HDACi can modulate the expression of approximately 5%–20% of genes [87]. For instance, treatment with quisinostat (a HDACi) in tongue squamous cell carcinoma cells resulted in an increase in ROS levels, a decrease in GPX4 protein expression, an increase in p53 protein expression, and changes in mitochondrial morphology and function were observed. These findings suggest the occurrence of ferroptosis [88]. Additionally, a study by Li F et al. demonstrated that quisinostat could activate p53 and promote ferroptosis by upregulating the acetylation of p53 [89]. Ines M L Wolf et al. found that the HDAC inhibitor SAHA specifically inhibits the expression of SLC7A11 transporter protein, which leads to increased ROS activity within glioma cells [64]. Furthermore, research conducted by Zhang T et al. revealed that vorinostat can reverse tumor cell resistance to ferroptosis by downregulating the expression of SLC7A11 [65].

Although HDAC inhibitors such as vorinostat, romidepsin, belinostat, panobinostat, and chidamide have been approved for the treatment of certain diseases, such as cutaneous T-cell lymphoma and multiple myeloma, these drugs have issues with low target specificity and low sensitivity to solid tumors. Therefore, researchers are developing the next-generation of HDAC inhibitors and exploring combination therapy strategies. Curcumin is a natural pan-HDAC inhibitor that has shown anti-tumor potential. However, its specific mechanism still requires further research. Curcumin has been found to reduce the vitality, GSH, and MMP levels in breast cancer cells while increasing ROS levels, apoptosis rates, and DNA damage. New drugs based on curcumin also show potential for the treatment of glioblastoma [90, 91]. In addition to exploring new HDAC inhibitors, researchers are also investigating combination therapies. For example, the combined use of HDAC inhibitors with other drugs like SASP can enhance the effect of ferroptosis. SASP itself is a known inducer of ferroptosis; when used in combination with vorinostat it can further promote this process by targeting SLC7A11 expression which is related to cancer cell insensitivity to HDAC inhibitors [92]. Furthermore Endri Karaj et al. have designed a new class of dual-mechanism hybrid molecules that can induce ferroptosis while inhibiting HDAC activity. These novel compounds are expected to become new types of anti-cancer agents that may reduce toxic side effects caused by ferroptosis [93]. Zille M et al and Paganoni S et al discovered that HDACi could induce ferroptosis in GBM without causing neurotoxic side effects [94, 95]. In summary, while research on the role of HDAC inhibitors in promoting ferroptosis has made progress, there remains a significant need to explore their complex mechanisms of action and to conduct a thorough assessment of the potential side effects in clinical applications.

## Conclusion

In recent years, ferroptosis has emerged as a novel mechanism of cell death and has become a prominent topic in the field of tumor research. Numerous researchers are dedicated to elucidating the mechanisms of ferroptosis and its association with various diseases, particularly its potential applications in tumor treatment. Despite offering new strategies for tumor treatment, there is currently a lack of clinical drug trials targeting ferroptosis, which constrains our ability to assess the safety and efficacy of ferroptosis-inducing agents in a clinical context [96]. Acetylation, an important post-translational modification, has been shown to impact the process of ferroptosis in studies. However, our current understanding of how acetylation affects ferroptosis is still limited, primarily focusing on individual key protein acetylation changes, and there is a need for further research to understand how acetylation influences protein interactions and its overall role in ferroptosis. This article reviews the role of histone deacetylases (HDACs) and their inhibitors in regulating the process of ferroptosis and anticipates future research directions. It is important to acknowledge the limitations inherent in this review, including potential publication bias towards positive findings, a possible overreliance on *in vitro* studies, and a lack of comprehensive clinical data to support the translational potential of HDAC inhibitors in ferroptosis. Additionally, the current body of research may not fully account for the heterogeneity of tumor types and stages, the individual variability among patients, or the long-term effects and safety profiles of HDAC inhibitors. Future studies should emphasize the role of acetylation in ferroptosis and explore its impact on tumor development and other diseases to establish a theoretical basis and experimental evidence for developing new treatment strategies. It is also crucial to address these limitations by employing rigorous study designs, seeking diverse patient populations, and conducting long-term follow-up studies to better understand the mechanisms and implications of ferroptosis in disease pathology. With continued deepening research efforts, we aim to gain a better understanding of the complex regulatory network involved in ferroptosis and make new breakthroughs in clinical treatment, while being mindful of the potential biases and gaps in the current literature.
